# Bilateral Wilms Tumor: A Surgical Perspective

**DOI:** 10.3390/children5100134

**Published:** 2018-09-24

**Authors:** Andrew J. Murphy, Andrew M. Davidoff

**Affiliations:** 1 Department of Surgery, St. Jude Children’s Research Hospital, Memphis, TN 38105, USA; andrew.davidoff@stjude.org; 2 Division of Pediatric Surgery, Department of Surgery, University of Tennessee Health Science Center, Memphis, TN 38105, USA

**Keywords:** bilateral Wilms tumor, nephron-sparing surgery

## Abstract

Historically, the management of bilateral Wilms tumor (BWT) was non-standardized and suffered from instances of prolonged chemotherapy and inconsistent surgical management which resulted in suboptimal renal and oncologic outcomes. Because of the risk of end-stage renal disease associated with the management of BWT, neoadjuvant chemotherapy and nephron-sparing surgery have been adopted as the guiding management principles. This management strategy balances acceptable oncologic outcomes against the risk of end-stage renal disease. A recent multi-institutional Children’s Oncology Group study (AREN0534) has confirmed the benefits of standardized 3-drug neoadjuvant chemotherapy and the utilization of nephron-sparing surgery in BWT patients; however, less than 50% of patients underwent bilateral nephron-sparing surgery. The coordination of neoadjuvant chemotherapy and the timing and implementation of bilateral nephron-sparing surgery are features of BWT management that require collaboration between oncologists and surgeons. This review discusses the surgical management strategy in the context of BWT disease biology, with an emphasis on timepoints during therapy at which surgical decision making can greatly impact this disease and minimize long-term toxicities.

## 1. Introduction

Wilms tumor (WT) is the most common kidney cancer in children and is the second most common pediatric extracranial solid tumor, affecting approximately 650 children in the United States annually [1]. Fascinatingly, 5–10% of patients present with bilateral Wilms tumor (BWT), which may present as synchronous or metachronous bilateral tumors [2]. The median age of diagnosis for BWT is 24 months, compared to 38 months for unilateral WT, which suggests a stronger underlying genetic predisposition [2]. This predisposition is also underscored by the increased incidence of BWT in patients with WT predisposition syndromes, including Beckwith–Wiedemann syndrome (BWS), Denys–Drash, and WAGR (Wilms tumor, aniridia, genitourinary anomalies, range of developmental anomalies) syndromes [3,4,5]. However, 85% of patients with BWS or WAGR syndromes who develop WT get unilateral disease [5,6]. Under Children’s Oncology Group (COG) protocols, unilateral WT is typically treated by up-front radical nephroureterectomy, with acceptable rates of long-term end-stage renal disease (<1%) [1,7]. Application of this strategy to patients with bilateral disease would render them anephric, and thus nephron-sparing approaches were developed and refined to preserve kidney function [8,9]. However, the long-term rate of end-stage renal disease for BWT patients still approaches 15%, necessitating strategies which achieve acceptable oncologic outcomes, but also preserve the maximum volume of functioning nephrons [10]. This approach should also be extended to syndromic patients who present with unilateral WT because of the risk of developing metachronous WT [11]. Historically, BWT management had not been standardized and has sometimes characterized by prolonged chemotherapy and inconsistent surgical management [12]. A recently completed multi-institutional COG study (AREN0534) has standardized the approach to neoadjuvant therapy and timing of surgical resection, resulting in great benefit to patients with BWT [11]. This review aims to detail the current, standardized approach to BWT in the context of disease biology and to emphasize the critical importance of surgical input in the longitudinal management of BWT patients. We will also emphasize critical aspects of the surgical and perioperative management of BWT.

## 2. Biology and Genetics

Surgeons must be familiar with the features of WT predisposition syndromes so that a nephron-sparing approach can be selected when there is a high risk of metachronous WT. Furthermore, differentiation of WT from nephrogenic rests (NRs), when possible, can avoid unnecessarily radical surgery. The presence of bilateral or multifocal disease or the association of WT with congenital anomalies suggests an underlying genetic predisposition for the development of WT. Overall, congenital anomalies are found in 9% of patients with unilateral WT and 33% of patients with BWT [13]. The earliest clue into the genetic basis of bilateral WT was derived from the observation that patients with WAGR syndrome often developed WT and had a higher incidence of BWT (15% of WAGR patients get BWT) [3]. These patients were also found to have a high prevalence of intralobar nephrogenic rests. It was later determined that WAGR was due to a germline deletion at 11p13 containing the *PAX6* and *WT1* genes [14,15,16]. The *WT1* transcription factor was later cloned and determined to be a potent tumor suppressor directly implicated in the development of WT [17]. Approximately 16% of BWT patients have a *WT1* germline mutation and 3% have affected family members [18]. BWT with *WT1* mutations are associated with early presentation (10 months versus 39 months for *WT1* wild-type patients) [18]. Tumors containing *WT1* mutations often also develop activating mutations in exon 3 of *CTNNB1*, resulting in Wnt pathway activation [19,20]. Tumors with this constellation of genetic mutations are prone to stromal differentiation during neoadjuvant therapy, which may preclude complete or significant regression of tumor volume. In fact, tumors with *WT1* mutation may paradoxically increase in size during therapy due to rhabdomyoblastic differentiation [2,21].

The prevalence of BWT is also higher in patients with genetic predisposition syndromes associated with somatic overgrowth including BWS and isolated hemihyperplasia [5]. BWS is a somatic overgrowth disorder characterized by macroglossia, hemihypertrophy, omphalocele, and a predisposition to develop embryonal tumors including WT, hepatoblastoma, embryonal rhabdomyosarcoma, and adrenocortical carcinoma. Approximately one-fifth of BWS patients who develop WT present with bilateral disease [22]. BWS is caused by a constellation of germline genetic or epigenetic aberrations at chromosome 11p15, which contains a cluster of imprinted genes. *IGF2*, which regulates organ growth in fetal development, is the most well-described imprinted gene at this locus. *IGF2* is normally imprinted such that it is only expressed from the paternal allele. The two most common mechanisms for loss of the normal imprinting pattern at this locus are uniparental isodisomy (copy neutral loss of heterozygosity by deletion of the maternal 11p15 and duplication of the paternal allele) or epigenetic loss of imprinting (selective gain of methylation at imprinting control region 1 adjacent to *H19* and *IGF2*), both of which result in biallelic expression of *IGF2* [23]. Patients with these molecular subtypes of BWS should undergo abdominal ultrasound every 3 months until age 8 [23]. Genetic testing for an 11p15 abnormality should be considered for patients exhibiting any features of BWS, hemihyperplasia, or bilateral or familial WT. BWT patients without syndromic features have been found to harbor germline constitutional abnormalities at 11p15 [24]. In nonsyndromic WT patients, somatic aberrations at these loci occur in approximately 70% of WT and with higher frequency in BWT. WT in patients with constitutional or somatic 11p15 anomalies are often associated with perilobar nephrogenic rests. BWS can present with a subtle, incomplete phenotype due to mosaic distribution of the 11p15 anomaly. Recent consensus guidelines detail the recommended molecular diagnostic measures, tumor-screening algorithms, and clinical management of patients with BWS [25].

### 2.1. Nephrogenic Rests and Nephroblastomatosis

**Figure 1:**
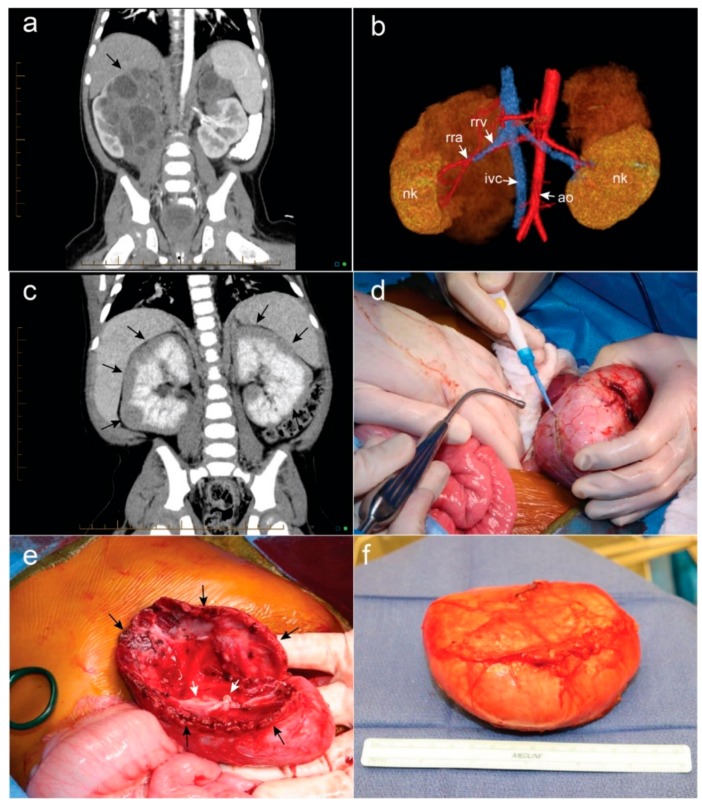
(**a**) Bilateral Wilms tumor (WT) demonstrating complex, ominous appearance of hilar right-sided tumor (black arrow) and more straightforward left-sided upper pole mass; (**b**) 3D rendering of bilateral tumors depicted in (**a**) assisted in delineating the vascular anatomy and bilateral nephron sparing surgery was possible. Nk = normal kidney, rra = right renal artery, rrv = right renal vein, ivc = inferior vena cava, ao = aorta; (**c**) bilateral diffuse hyperplastic perilobar nephroblastomatosis is depicted with rind-like peripheral expansion of nephrogenic rests (black arrows); (**d**) a marginal resection of bilateral WT is performed by developing the plane between the encapsulated tumor and normal renal parenchyma; (**e**) after nephron sparing surgery is performed, the collecting system (white arrows) is closed with monofilament suture. If possible, the redundant edges of normal renal parenchyma (black arrows) can be approximated over the raw surface of the kidney; (**f**) Appearance of the encapsulated WT following surgical resection.

The majority of BWTs are associated with NRs, which are undifferentiated islands of embryonic metanephric blastema that are thought to be premalignant WT precursor lesions. Perilobar NRs are associated with 11p15 anomalies, while intralobar NRs are associated with variants in *WT1*/11p13. A single patient can have both perilobar and intralobar NRs (combined NRs). NRs are associated with higher rates of metachronous WT development. BWT exhibit NRs in 84% of cases, while unilateral WT are associated with NRs in 38% of cases. BWT patients exhibit a higher proportion of perilobar NRs (52%) when compared to intralobar or combined NRs (32%) [13,26]. NRs are most often microscopic lesions that are not detected on imaging. However, expansile, multiple, or diffuse NRs (nephroblastomatosis) may be detected on imaging. Differentiating nephroblastomatosis from WT at the time of diagnosis is very difficult, but critical in deciding the correct treatment plan. This is particularly important in cases of unilateral WT with contralateral nephroblastomatosis, or cases of bilateral nephroblastomatosis, both of which can be mistaken for cases of BWT. WTs are generally surrounded by a fibrous capsule that separates the lesion from the normal kidney while NRs are not. However, percutaneous biopsy does not reliably distinguish nephroblastomatosis from WT, as the interface of the WT and adjacent normal kidney is difficult to reliably capture. Molecular analysis is also unreliable in a clinical setting, as NRs are WT precursor lesions and therefore contain genetic variants that are also present in WT. Therefore, imaging is the most reliable, albeit an imperfect way to distinguish WT from NRs. Prior to chemotherapy, NRs are homogeneous in appearance both before and after contrast agent administration on a computerized tomography (CT) scan or magnetic resonance imaging (MRI). After chemotherapy, MRI can distinguish NRs from WT because viable WT enhance on T2 and STIR sequences while NR do not. WT should be strongly suspected when the lesion in question enhances, is spherical, and exceeds 1 cm in diameter. Hyperplastic perilobar nephrogenic rests (HPN) refers to a proliferation of NRs that is multifocal and often bilateral. Included in the category of HPN is a subtype known as diffuse hyperplastic perilobar nephroblastomatosis (DHPN), which describes a pattern of expansile NRs that extend along the periphery of the kidney. The characteristic appearance of DHPN on CT or MRI scans is a homogenous, rind-like expansion of tissue on the periphery of the kidney (Figure 1). The appearance maintains the overall architecture of the kidney and can look like a “giant kidney” on imaging. This is generally distinguishable from the round, heterogeneous appearance of WT which may distort the architecture of the kidney and result in a “claw sign” in the instance of large lesions (Figure 1).

In the absence of WT, the management of HPN is non-operative. Chemotherapy is recommended for HPN as a means of decreasing the cellular mass predisposed to the development of WT. Observation without initiation of therapy is regarded as a non-standard option because the rate of malignant transformation appears to be quite high and to persist for years after initial diagnosis. The current recommended treatment for HPN is combination chemotherapy with actinomycin-D and vincristine (EE-4A regimen used for the treatment of stage I/II WT), although treatment duration is sometimes extended due to the variable timing of response [27]. However, prolonged courses of chemotherapy may select for anaplastic transformation [28], which is much more common in patients with HPN who develop WT (32%) than in patients with sporadic WT [29]. Perlman et al. published a large series of 52 patients who presented with HPN from the National Wilms Tumor Study, 49 (94%) of whom had bilateral disease. Forty-six (88%) patients presented with DHPN characterized by a “rind-like” appearance on imaging [29]. Three (5.8%) patients in this series were observed without initiation of chemotherapy and all three developed WT between 4–10 months after diagnosis (none died from disease). Of the remaining 49 patients who received therapy, 33 patients underwent biopsy and initiation of chemotherapy and 16 patients underwent unilateral nephrectomy followed by the initiation of chemotherapy. Of the 33 patients who underwent biopsy and chemotherapy, 18 developed WT and 15 did not (3 of these WT patients died from disease; 2 developed diffuse anaplasia). Of the 16 patients who underwent unilateral nephrectomy followed by chemotherapy, 13 patients did not develop a subsequent lesion and 3 patients developed WT (none died from disease). Because patients with HPN are predisposed to the subsequent development of WT, even in radiographically normal areas of kidney, a 3-month surveillance imaging interval is recommended, and the length of follow-up should be continued for a minimum of 7 years following initial diagnosis. Any suspected tumors increasing in size during chemotherapy in HPN patients should be promptly resected, if feasible, as there is a stronger likelihood of diffuse anaplasia in this group. Thus, initiation of chemotherapy, close imaging follow-up, and a higher index of suspicion for the development of diffuse anaplasia are the guiding principles of HPN management [29]. Up-front nephrectomy should be avoided, but timely nephron-sparing surgery (NSS) should be employed when there is suspicion for the development of WT, which will occur in most cases.

When a patient with HPN develops a focus of Wilms tumor, a nephron-sparing approach is generally recommended given the predisposition for metachronous disease. During the conduct of this operation, the surgeon should focus on delineating the WT from surrounding tissue, whether that be NRs or normal kidney. The distinction between normal kidney and NRs cannot always be readily determined intraoperatively. Biopsies from areas of uncertainty can be obtained, however definitive diagnosis of nephrogenic rests typically requires an adjacent area of normal kidney and requires permanent section.

### 2.2. Diagnosis and Neoadjuvant Therapy

The diagnosis of BWT is typically confirmed by the presence of bilateral renal masses, in an appropriately aged child, on ultrasound followed by contrast-enhanced CT abdomen/pelvis. A CT chest should also be performed to evaluate for pulmonary metastases at diagnosis, the most common site of metastatic disease in children with WT. Because other pediatric renal tumors are almost never bilateral, neoadjuvant chemotherapy for presumed BWT should be initiated without first performing a biopsy of any of the tumors [11]. In the recently completed first multicenter study specifically for BWT patients, the COG AREN0534 trial, intensification of neoadjuvant chemotherapy to three-drugs with vincristine, actinomycin-D, and doxorubicin (VAD) resulted in improved 3-year event free and overall survival compared to historical BWT patients treated on the NWTS-5 protocol who were often treated with only vincristine and actinomycin-D [11,30]. The rationale for up-front intensification of therapy was to achieve improved tumor response to facilitate bilateral nephron-sparing surgery. The described surgical management algorithm is identical for patients with syndromic or non-syndromic BWT. For patients with syndromic unilateral WT, this management algorithm is also followed due to the risk of metachronous WT. This management algorithm is not applicable to patients with unilateral, non-syndromic WT. 

**Figure 2:**
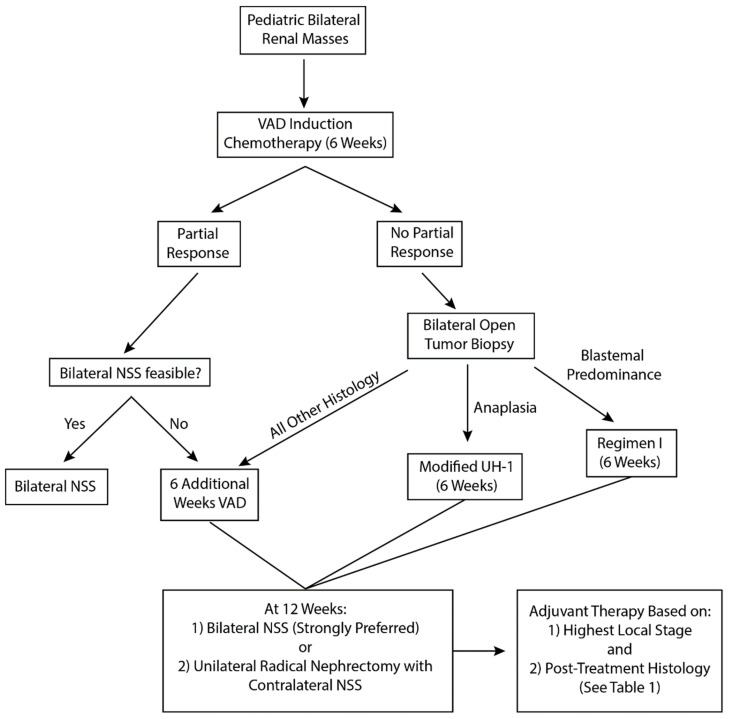
Surgical management algorithm for bilateral Wilms tumor. NSS—Nephron-sparing surgery; VAD—vincristine, actinomycin-D, doxorubicin; Revised UH-1—vincristine, dactinomycin, doxorubicin, cyclophosphamide, carboplatin, etoposide; Regimen I—vincristine, dactinomycin, doxorubicin, cyclophosphamide, etoposide.

The feasibility of bilateral nephron-sparing surgery should be assessed after six weeks of VAD therapy by contrast-enhanced CT scan of the abdomen/pelvis (Figure 2). If more than 50% volume reduction has been achieved for all tumors, but nephron sparing surgery is still not feasible, neoadjuvant VAD should be continued for six more weeks. Surgical resection should be performed regardless of tumor status at the 12-week timeframe. If less than 50% volume reduction has been achieved after the six initial weeks of therapy for any tumor, and bilateral nephron-sparing surgery is still not feasible, open surgical biopsy of all tumors should be performed to assess for the possibility of alternative histologies such as diffuse anaplasia, blastemal predominance after neoadjuvant therapy, differentiated tumor without remaining viable/proliferative elements, or an alternate diagnosis (exceedingly uncommon). Core biopsy often misses the presence of diffuse anaplasia, which could be responsible for treatment resistance, and should thus be avoided in this treatment algorithm [31]. The histology of the tumor at the time of open biopsy will direct 6 weeks of additional chemotherapy (VAD, regimen I, or modified UH-1; Figure 2). Administration of neoadjuvant therapy beyond 12 weeks should not occur, as it may impart significant long-term toxicity without discernible oncologic benefit and will not facilitate an easier operation. Furthermore, prolonged neoadjuvant chemotherapy has been associated with anaplastic transformation in BWT, particularly in patients treated for DHPN [12,28].

If a complete response for both kidney tumors is observed for patients without metastatic disease after 6-weeks of VAD, then adjuvant regimen EE-4A is instituted (actinomycin-D, vincristine). For BWT patients with metastatic disease who exhibit a complete response at the primary sites after 6-weeks of VAD, adjuvant regimen DD-4A (vincristine, actinomycin-D, and doxorubicin) is instituted. Otherwise, the described surgical management algorithm is identical for patients with metastatic and non-metastatic disease. A discussion of the management of pulmonary metastases or other metastatic sites is beyond the scope of this review.

### 2.3. Surgical Management

In our experience of more than 60 operations for BWT (42 patients have published long-term follow-up), approximately 90% of patients can undergo successful bilateral nephron-sparing surgery, despite occasionally ominous pre-treatment imaging (Figure 1) [9,32,33]. The surgical anatomy of BWT is best delineated by a preoperative, multiphase contrast-enhanced CT scan of the abdomen and pelvis. Three-dimensional reconstruction of cross-sectional images may help the surgeon better understand the relationship of the tumor to the relevant renal vasculature and collecting system [34]. A three-dimensional appreciation of the tumor’s position relative to the surrounding normal renal parenchyma may also facilitate the intraoperative approach to the tumor (Figure 1). Some authors have experimented with 3D printed models of BWT anatomy to facilitate operative planning [35]. There are no accepted, defined criteria for preoperative determination of bilateral NSS feasibility. Future studies aim to determine imaging criteria associated with positive margins or the need for radical nephrectomy. However, small tumor size, peripheral or polar location of the mass, and lack of invasion or encasement of renal vessels are intuitive, encouraging features for a nephron-sparing approach. On the other hand, large tumor size, central location, and proximity to the renal vessels should not be regarded as absolute contraindications for NSS. We submit that these “ominous” imaging features warrant referral to an experienced center for assessment of bilateral NSS rather than committing to radical nephrectomy. BWT with associated tumor thrombus (renal vein, inferior vena cava (IVC), atrial extension) will typically require radical nephrectomy for en bloc resection of the tumor thrombus unless there is complete regression of the tumor thrombus during neoadjuvant chemotherapy. 

A laparotomy is performed through a bilateral subcostal incision to gain access to both kidneys and associated tumors. Full medial visceral rotation is performed for large tumors, while mobilization of the colon or colon and duodenum may suffice for smaller tumors, as is often the case after neoadjuvant chemotherapy. The kidney and tumor(s) are completely mobilized. The main renal artery and vein are dissected and encircled with vessel loops to be used for vascular control in cases of temporary bleeding. Careful handling of the vascular pedicle is critically important, because traction injury with vascular thrombosis or spasm can occur, especially in very young patients. We use manual compression of the renal hilum rather than vessel loops if the hilum is readily accessible. We do not routinely utilize vascular clamps or preemptively occlude the renal vasculature during the parenchymal dissection. We do not routinely ligate branches of the renal artery or vein outside the renal parenchyma prior to beginning transection. We often perform an enucleation technique (marginal resection) focused on resecting the tumor with an intact capsule to preserve the greatest volume of residual normal renal parenchyma. The dissection is performed with a right-angle clamp and electrocautery (Figure 1). All discrete multifocal lesions are usually independently resected during the operation. If the WT is arising in an extensive area of nephrogenic rests, the tumor can be resected and the rests biopsied to rule out WT in these areas. Any multifocal lesions that have completely responded on preoperative imaging should be biopsied intraoperatively if a visible scar is noted at the site of the original multifocal lesion. Focal areas of bleeding corresponding to intraparenchymal renal vessels are controlled with prolene suture. Violations of the renal collecting system are closed with a monocryl suture (Figure 1). Hemostasis at the cut edge of renal parenchyma can be facilitated by applying temporary pressure, followed by argon beam coagulation, and placement of an absorbable topical hemostatic agent. In about half of cases, there is sufficient, redundant renal parenchyma to close over the collecting system with silk suture (Figure 1). We do not routinely place ureteral stents or transperitoneal drains unless there is intraoperative concern about tension on the collecting system closure. In general, post-operative surgical complications are more common after nephron-sparing surgery when compared to radical nephrectomy. In the event of postoperative urinoma, percutaneous drains can be placed using image guidance and ureteral stents placed by cystoscopy. Paraaortic, aortocaval, paracaval, and parailiac lymph nodes are sampled for local staging of each tumor when present.

We utilize this marginal resection approach to nephron-sparing surgery for most lesions, including large lesions or those with hilar involvement (Figure 1) [9]. Alternatively, techniques that involve parenchymal renal transection, including longitudinal partial nephrectomy, have been described to approach hilar lesions [34]. Although we have not found renal cooling or vascular clamping to be necessary measures, Millar et al. reported a series of 23 patients in which this technique was commonly used with good long-term preservation of renal function [36]. We have not found intraoperative frozen section to be necessary. 

The local stage of each tumor (margins, rupture, lymph node positivity) should be assessed and documented by the pathologist. The post-treatment histology risk (originally derived from International Society of Pediatric Oncology (SIOP) protocols in which up-front chemotherapy is utilized for nearly all WT—unilateral or bilateral) should be assessed based on the percentage of necrosis or blastemal predominance or frank anaplasia in the resected specimens. Both the local stage and post-treatment histology will guide the selection of adjuvant therapies (Table 1) [37].

Nephron-sparing surgery should also be performed for unilateral WT in patients with a solitary kidney, horseshoe kidney, or WT predisposition syndrome that makes the patient prone to metachronous bilateral disease or eventual renal failure.

### 2.4. Standardized Documentation of Nephron-Sparing Surgery

A review published by the SIOP Renal Tumors Study Group noted three critical deficiencies in surgical operative notes, pathology reports, and the published literature pertaining to NSS: (1) definitions of the surgical approach are unclear, confusing, and highly variable; (2) the subjective surgical resection margin is often omitted from reports; and (3) an assessment of the remaining volume of renal parenchyma is often not included. Therefore, consensus recommendations were developed by SIOP pathologists and surgeons for the reporting of NSS, whether for bilateral or unilateral WT (Table 2) [38]. The theoretical, but unproven, advantage of performing a partial nephrectomy over an enucleation is a lower incidence of positive pathological margins. However, this would come at the cost of leaving less remaining renal parenchyma. This standardized reporting could more consistently determine associations between surgical technique and margin status or long-term renal outcomes [38].

### 2.5. Difficult Surgical Scenarios

#### 2.5.1. Positive Margins

To preserve maximal residual renal parenchyma, a marginal resection or enucleation technique is often used for bilateral nephron-sparing surgery [33]. Despite utilizing a marginal resection technique, surgical margins are very often negative because WTs tend to grow within a surrounding fibrous capsule that does not contain malignant cells. However, a positive margin at the time of nephron-sparing surgery results in local stage 3 disease, for which flank radiotherapy is generally recommended, according to the AREN0534 protocol [11]. In patients treated at St. Jude Children’s Research Hospital, a positive margin in BWT patients undergoing NSS was not associated with local disease relapse, with most patients receiving radiation therapy [32,39]. However, a positive margin in a kidney tumor with diffuse anaplasia confers a poor prognosis and thus surgeons must have high index of suspicion in tumors that have been completely unresponsive to induction therapy [32]. In cases of a positive margin in tumors with diffuse anaplasia, completion nephrectomy followed by flank irradiation should be strongly considered. A margin which contains nephrogenic rests has no bearing on patient prognosis or local disease relapse.

#### 2.5.2. Local Relapse

The estimated rate of local recurrence for WT patients is between 8.5% and 13.9% [40,41]. Although all patients with WT must be monitored closely for local tumor recurrence, the multifocality of BWT, the young age at which patients develop primary tumors, and the increased prevalence of syndromes associated with renal compromise make the treatment of tumor recurrence in patients with BWT especially challenging. In BWT patients, local disease relapse occurs with similar frequency whether there was a positive margin associated with the original surgical resection or not [39]. Because BWT patients often have multifocal disease, local relapse may often be due to small multifocal tumors that are unrecognized at the time of the original surgery rather than true recurrence of a resected tumor [39]. Positive margins are more frequent after redoing nephron-sparing surgery when compared to initial nephron-sparing surgery because of increased technical difficulty in delineating the plane between the tumor and normal kidney in this setting [42]. Local relapse with the development of diffuse anaplasia confers an exceedingly poor prognosis in this patient population [32]. A biopsy of local relapse is typically unnecessary if there is evidence of increasing volume on imaging. However, a biopsy may be required if there is persistent uncertainty regarding the differentiation between disease relapse and postoperative changes/scar. Salvage chemotherapy is typically instituted prior to redoing nephron-sparing surgery, unless the relapse is suspected to be from a missed lesion and the redone nephron-sparing surgery is deemed to be straightforward. 

#### 2.5.3. Diffuse Anaplasia

Histology remains the most important component of WT risk stratification, even more than stage. Diffuse anaplasia (unfavorable histology) occurs in 6% of WT patients, is associated with significant treatment resistance, and accounts for 50% of WT deaths. Diffuse anaplasia is defined by the multifocal presence of large hyperchromatic nuclei with abnormal multipolar mitoses. The development of diffuse anaplasia is likely a late event in tumorigenesis that is related to selection of a *TP53* mutant clone within an initially favorable histology WT [43]. In BWT with anaplasia, there is usually discordant histology between bilateral tumors. In a series of 27 patients with BWT and diffuse anaplasia treated on NWTS-4 protocol, only 4 had diffuse anaplasia in both kidney tumors. Furthermore, core needle biopsy failed to identify diffuse anaplasia in all biopsied patients who were eventually found to have anaplastic disease [31]. Diffuse anaplasia is more common in BWT than unilateral WT [11]. Diffuse anaplasia is found in 32% of WT that develop in patients with DHPN [28]. In our series of 42 patients who underwent surgery for BWT, there were 6 deaths. Two of these patients were found to have diffuse anaplasia in their original surgical resection specimens and three had diffuse anaplasia in local disease relapse specimens [32]. Given these results, we do not advocate for a nephron-sparing approach when anaplastic histology is known preoperatively by biopsies of tumors that fail to demonstrate volumetric regression during the first six weeks of neoadjuvant therapy or in instances of anaplasia detected in biopsy of local recurrence. If diffuse anaplasia is not known by biopsy or strongly suspected preoperatively, it may be discovered from permanent pathology after NSS. If a positive margin with diffuse anaplasia is discovered in this setting, we advocate for completion nephrectomy. If anaplasia is detected, but the surgical margins are negative, we do not advocate for completion of the nephrectomy. 

#### 2.5.4. Repeat Nephron-Sparing Surgery

Of 42 patients treated for synchronous BWT in our series, 39 (92.9%) underwent initial bilateral NSS. Thirteen (31.0%) patients had at least one surgical specimen with a positive margin at initial operation; three more patients had a specimen with a positive margin at a subsequent surgery (see below) [32]. Three patients required early repeat NSS within four months of their initial surgery for suspected residual tumor; 7 patients (16.7%) experienced local tumor recurrence (greater than four months from initial surgery); 6 of these 7 patients were managed with repeat NSS and 1 was managed with completion unilateral nephrectomy. Thus, repeat NSS is a viable option for both residual tumor and local relapse after bilateral NSS for BWT. The presence of diffuse anaplasia in a surgical resection specimen should be regarded as a contraindication for repeat NSS [32].

### 2.6. Long-Term Follow-Up/Renal Outcomes

Renal function outcomes were assessed in 36 of 42 surviving patients from our institutional series with a median follow up time of 3.7 years (range: 0.03–13.4 years). No patients required chronic dialysis or kidney transplantation. Eleven (30.6%) patients were on an antihypertensive medication at last follow-up. Of patients not taking anti-hypertensives, two were noted to have persistent systolic or diastolic blood pressure readings which exceeded the 95th percentile for their age group and 5 other patients were noted to have readings between the 90th and 95th percentiles for their age group, considered pre-hypertensive. Five patients had ≥1+ proteinuria. 8.3% of patients had stage-2 chronic kidney disease as defined by an estimated glomerular filtration rate (eGFR) less than 90 mL/min/1.73 m^2^ and ≥1+ proteinuria [32,44]. Thus, acceptable long-term renal outcomes are achievable with a bilateral nephron-sparing approach.

### 2.7. Renal Transplantation for Bilateral Wilms Tumor

Bilateral nephrectomy may be required in BWT patients because of disease relapse or complications of surgical or medical therapy, necessitating hemodialysis and eventual renal transplantation. Also, bilateral nephrectomy will likely eventually be required for WT patients with Denys–Drash syndrome, who eventually develop nephropathy and end-stage renal disease. Transplantation has historically been delayed until 1–2 years after cancer therapy because most WT relapses occur within 2 years of diagnosis [45]. However, more recent data show WT patients, even those who underwent early transplant, have outcomes similar to other renal transplant patients [46]. Given the morbidity and mortality associated with chronic pediatric dialysis, consideration of earlier transplantation should be made in WT patients with end stage renal disease and no evidence of cancer.

## 3. Conclusions

The current treatment strategy for BWT balances acceptable oncologic outcomes with the goal of maintaining the maximum amount of functioning renal parenchyma. It is imperative that the pediatric surgeon be intimately aware of the treatment algorithm for BWT when managing such patients. This algorithm is characterized by avoidance of initial tumor biopsy, three-drug neoadjuvant chemotherapy (VAD), assessment of tumor volumetric response at 6 weeks, a decision whether to continue VAD, perform an open biopsy, or change chemotherapy for a subsequent six weeks, and then to perform surgical resection in all cases at 12 weeks of therapy. Close collaboration between pediatric surgeons and the multidisciplinary oncology team is necessary to negotiate this complex algorithm and to maximize the chances that BWT patients have optimal long-term oncologic and renal outcomes.

## Figures and Tables

**Table 1:** Determination of adjuvant therapy after surgical resection of bilateral Wilms tumor [11].

Histology	Stage	Adjuvant Regimen
Completely necrotic	I–II	EE-4A
Intermediate risk	I	EE-4A
Intermediate risk	II	DD-4A
Intermediate risk	III–IV	DD-4A + XRT
Blastemal predominant	I	DD-4A
Diffuse anaplasia	I	DD-4A + XRT
Completely necrotic	III–IV	DD-4A + XRT
Focal anaplasia	I–III	DD-4A + XRT
Blastemal predominant	II	Regimen I
Blastemal predominant	III–IV	Regimen I + XRT
Focal anaplasia	IV	Revised UH-1 + XRT
Diffuse anaplasia	II–IV	Revised UH-1 + XRT

EE-4A—vincristine and dactinomycin; DD-4A—vincristine, dactinomycin, doxorubicin; regimen I—vincristine, dactinomycin, doxorubicin, cyclophosphamide, etoposide; revised UH-1 vincristine, dactinomycin, doxorubicin, cyclophosphamide, carboplatin, etoposide; XRT—radiation therapy (flank radiotherapy in most cases). Reproduced with permission from reference [11]: Ehrlich et al., *Annals of Surgery*; published by Wolters Kluwer Health, Inc, 2017.

**Table 2:** International Society of Pediatric Oncology (SIOP) standardized reporting system for nephron sparing surgery [38].

Reporting Component Format: *NSS(X)-SRM(n)-PRM(n)-RRP(n%)*	Description
1. Surgical Technique	
a. NSS(A)—partial nephrectomy	Resection of the tumor with a rim of normal renal parenchyma
b. NSS(B)—enucleation (marginal resection)	Resection of the tumor without a rim of normal renal parenchyma
2. Surgical Resection Margin (SRM)	Surgeon’s impression of resection margin
a. Intact pseudo-capsule = (0)	
b. Doubt intact pseudo-capsule = (1)	
c. Definite tumor breach = (2)	
3. Pathological Resection Margin (PRM)	Microscopic resection margin on permanent pathology
a. Rim of normal renal parenchyma on resection margin (=0)	Exception for nephroblastomatosis
b. Intact pseudo-capsule along resection margin (=1)	
c. Tumor breach (=2)	
4. Remaining Renal Parenchyma (RRP) = (*n*%)	Surgeon’s assessment of the percentage of remaining normal renal parenchyma

NSS—nephron-sparing surgery, SRM—surgical resection margin, PRM—pathological resection margin, RRP—remaining renal parenchyma. Table is adapted from reference [38].

## References

[1] Davidoff A.M. (2012). Wilms tumor. Adv. Pediatr..

[2] Charlton J., Irtan S., Bergeron C., Pritchard-Jones K. (2017). Bilateral Wilms tumour: A review of clinical and molecular features. Expert Rev. Mol. Med..

[3] Breslow N.E., Norris R., Norkool P.A., Kang T., Beckwith J.B., Perlman E.J., Ritchey M.L., Green D.M., Nichols K.E., National Wilms Tumor Study Group (2003). Characteristics and outcomes of children with the wilms tumor-aniridia syndrome: A report from the national Wilms tumor study group. J. Clin. Oncol..

[4] Pelletier J., Bruening W., Kashtan C.E., Mauer S.M., Manivel J.C., Striegel J.E., Houghton D.C., Junien C., Habib R., Fouser L. (1991). Germline mutations in the Wilms’ tumor suppressor gene are associated with abnormal urogenital development in denys-drash syndrome. Cell.

[5] Porteus M.H., Narkool P., Neuberg D., Guthrie K., Breslow N., Green D.M., Diller L. (2000). Characteristics and outcome of children with beckwith-wiedemann syndrome and Wilms’ tumor: A report from the national wilms tumor study group. J. Clin. Oncol..

[6] Huff V. (1998). Wilms tumor genetics. Am. J. Med. Genet..

[7] Interiano R.B., Delos Santos N., Huang S., Srivastava D.K., Robison L.L., Hudson M.M., Green D.M., Davidoff A.M. (2015). Renal function in survivors of nonsyndromic Wilms tumor treated with unilateral radical nephrectomy. Cancer.

[8] Cozzi D.A., Zani A. (2006). Nephron-sparing surgery in children with primary renal tumor: Indications and results. Semin. Pediatr. Surg..

[9] Davidoff A.M., Giel D.W., Jones D.P., Jenkins J.J., Krasin M.J., Hoffer F.A., Williams M.A., Dome J.S. (2008). The feasibility and outcome of nephron-sparing surgery for children with bilateral wilms tumor. The st jude children’s research hospital experience: 1999–2006. Cancer.

[10] Breslow N.E., Collins A.J., Ritchey M.L., Grigoriev Y.A., Peterson S.M., Green D.M. (2005). End stage renal disease in patients with Wilms tumor: Results from the national wilms tumor study group and the united states renal data system. J. Urol..

[11] Ehrlich P., Chi Y.Y., Chintagumpala M.M., Hoffer F.A., Perlman E.J., Kalapurakal J.A., Warwick A., Shamberger R.C., Khanna G., Hamilton T.E. (2017). Results of the first prospective multi-institutional treatment study in children with bilateral Wilms tumor (aren0534): A report from the children’s oncology group. Ann. Surg..

[12] Shamberger R.C., Haase G.M., Argani P., Perlman E.J., Cotton C.A., Takashima J., Green D.M., Ritchey M.L. (2006). Bilateral Wilms’ tumors with progressive or nonresponsive disease. J. Pediatr. Surg..

[13] Vujanic G.M., Apps J.R., Moroz V., Ceroni F., Williams R.D., Sebire N.J., Pritchard-Jones K. (2017). Nephrogenic rests in Wilms tumors treated with preoperative chemotherapy: The UK SIOP Wilms tumor 2001 trial experience. Pediatr. Blood Cancer.

[14] Pritchard-Jones K., Fleming S., Davidson D., Bickmore W., Porteous D., Gosden C., Bard J., Buckler A., Pelletier J., Housman D. (1990). The candidate Wilms’ tumour gene is involved in genitourinary development. Nature.

[15] Compton D.A., Weil M.M., Jones C., Riccardi V.M., Strong L.C., Saunders G.F. (1988). Long range physical map of the wilms’ tumor-aniridia region on human chromosome 11. Cell.

[16] Pelletier J., Bruening W., Li F.P., Haber D.A., Glaser T., Housman D.E. (1991). WT1 mutations contribute to abnormal genital system-development and hereditary Wilms-tumor. Nature.

[17] Huff V. (2011). Wilms’ tumours: About tumour suppressor genes, an oncogene and a chameleon gene. Nat. Rev. Cancer.

[18] Hu M., Fletcher J., McCahon E., Catchpoole D., Zhang G.Y., Wang Y.M., Algar E.M., Alexander S.I. (2013). Bilateral Wilms tumor and early presentation in pediatric patients is associated with the truncation of the Wilms tumor 1 protein. J. Pediatr..

[19] Koesters R., Niggli F., von Knebel Doeberitz M., Stallmach T. (2003). Nuclear accumulation of beta-catenin protein in Wilms’ tumours. J. Pathol..

[20] Maiti S., Alam R., Amos C.I., Huff V. (2000). Frequent association of beta-catenin and WT1 mutations in Wilms tumors. Cancer Res..

[21] Royer-Pokora B., Beier M., Brandt A., Duhme C., Busch M., de Torres C., Royer H.D., Mora J. (2018). Chemotherapy and terminal skeletal muscle differentiation in WT1-mutant Wilms tumors. Cancer Med..

[22] Green D.M., Breslow N.E., Beckwith J.B., Norkool P. (1993). Screening of children with hemihypertrophy, aniridia, and beckwith-wiedemann syndrome in patients with Wilms tumor: A report from the national wilms tumor study. Med. Pediatr. Oncol..

[23] Mussa A., Molinatto C., Baldassarre G., Riberi E., Russo S., Larizza L., Riccio A., Ferrero G.B. (2016). Cancer risk in beckwith-wiedemann syndrome: A systematic review and meta-analysis outlining a novel (EPI)genotype specific histotype targeted screening protocol. J. Pediatr..

[24] Scott R.H., Douglas J., Baskcomb L., Huxter N., Barker K., Hanks S., Craft A., Gerrard M., Kohler J.A., Levitt G.A. (2008). Constitutional 11p15 abnormalities, including heritable imprinting center mutations, cause nonsyndromic Wilms tumor. Nat. Genet..

[25] Brioude F., Kalish J.M., Mussa A., Foster A.C., Bliek J., Ferrero G.B., Boonen S.E., Cole T., Baker R., Bertoletti M. (2018). Expert consensus document: Clinical and molecular diagnosis, screening and management of beckwith-wiedemann syndrome: An international consensus statement. Nat. Rev. Endocrinol..

[26] Lange J., Peterson S.M., Takashima J.R., Grigoriev Y., Ritchey M.L., Shamberger R.C., Beckwith J.B., Perlman E., Green D.M., Breslow N.E. (2011). Risk factors for end stage renal disease in non-WT1-syndromic wilms tumor. J. Urol..

[27] Stabouli S., Printza N., Dotis J., Matis A., Koliouskas D., Gombakis N., Papachristou F. (2014). Perilobar nephroblastomatosis: Natural history and management. Case Rep. Pediatr..

[28] Furtwangler R., Schmolze M., Graber S., Leuschner I., Amann G., Schenk J.P., Niggli F., Kager L., von Schweinitz D., Graf N. (2014). Pretreatment for bilateral nephroblastomatosis is an independent risk factor for progressive disease in patients with stage V nephroblastoma. Klin. Padiatr..

[29] Perlman E.J., Faria P., Soares A., Hoffer F., Sredni S., Ritchey M., Shamberger R.C., Green D., Beckwith J.B., National Wilms Tumor Study Group (2006). Hyperplastic perilobar nephroblastomatosis: Long-term survival of 52 patients. Pediatr. Blood Cancer.

[30] Hamilton T.E., Ritchey M.L., Haase G.M., Argani P., Peterson S.M., Anderson J.R., Green D.M., Shamberger R.C. (2011). The management of synchronous bilateral Wilms tumor: A report from the national wilms tumor study group. Ann. Surg..

[31] Hamilton T.E., Green D.M., Perlman E.J., Argani P., Grundy P., Ritchey M.L., Shamberger R.C. (2006). Bilateral Wilms’ tumor with anaplasia: Lessons from the national Wilms’ tumor study. J. Pediatr. Surg..

[32] Davidoff A.M., Interiano R.B., Wynn L., Delos Santos N., Dome J.S., Green D.M., Brennan R.C., McCarville M.B., Krasin M.J., Kieran K. (2015). Overall survival and renal function of patients with synchronous bilateral Wilms tumor undergoing surgery at a single institution. Ann. Surg..

[33] Kieran K., Davidoff A.M. (2015). Nephron-sparing surgery for bilateral Wilms tumor. Pediatr. Surg. Int..

[34] Fuchs J., Szavay P., Seitz G., Handgretinger R., Schafer J.F., Warmann S.W. (2011). Nephron sparing surgery for synchronous bilateral nephroblastoma involving the renal Hilus. J. Urol..

[35] Giron-Vallejo O., Garcia-Calderon D., Ruiz-Pruneda R., Cabello-Laureano R., Domenech-Abellan E., Fuster-Soler J.L., Ruiz-Jimenez J.I. (2018). Three-dimensional printed model of bilateral Wilms tumor: A useful tool for planning nephron sparing surgery. Pediatr. Blood Cancer.

[36] Millar A.J., Davidson A., Rode H., Numanoglu A., Hartley P.S., Desai F. (2011). Nephron-sparing surgery for bilateral Wilms’ tumours: A single-centre experience with 23 cases. Afr. J. Paediatr. Surg..

[37] Dome J.S., Graf N., Geller J.I., Fernandez C.V., Mullen E.A., Spreafico F., Van den Heuvel-Eibrink M., Pritchard-Jones K. (2015). Advances in Wilms tumor treatment and biology: Progress through international collaboration. J. Clin. Oncol..

[38] Godzinski J., Graf N., Audry G. (2014). Current concepts in surgery for Wilms tumor—The risk and function-adapted strategy. Eur. J. Pediatr. Surg..

[39] Kieran K., Williams M.A., Dome J.S., McGregor L.M., Krasin M.J., Davidoff A.M. (2013). Margin status and tumor recurrence after nephron-sparing surgery for bilateral Wilms tumor. J. Pediatr. Surg..

[40] Shamberger R.C., Guthrie K.A., Ritchey M.L., Haase G.M., Takashima J., Beckwith J.B., D’Angio G.J., Green D.M., Breslow N.E. (1999). Surgery-related factors and local recurrence of Wilms tumor in national Wilms tumor study 4. Ann. Surg..

[41] Dome J.S., Liu T., Krasin M., Lott L., Shearer P., Daw N.C., Billups C.A., Wilimas J.A. (2002). Improved survival for patients with recurrent wilms tumor: The experience at St. Jude children’s research hospital. J. Pediatr. Hematol. Oncol..

[42] Kieran K., Williams M.A., McGregor L.M., Dome J.S., Krasin M.J., Davidoff A.M. (2014). Repeat nephron-sparing surgery for children with bilateral Wilms tumor. J. Pediatr. Surg..

[43] Ooms A.H., Gadd S., Gerhard D.S., Smith M.A., Guidry Auvil J.M., Meerzaman D., Chen Q.R., Hsu C.H., Yan C., Nguyen C. (2016). Significance of TP53 mutation in wilms tumors with diffuse anaplasia: A report from the children’s oncology group. Clin. Cancer Res..

[44] Interiano R.B., McCarville M.B., Santos N.D., Mao S., Wu J., Dome J.S., Kieran K., Williams M.A., Brennan R.C., Krasin M.J. (2017). Comprehensive renal function evaluation in patients treated for synchronous bilateral Wilms tumor. J. Pediatr. Surg..

[45] Kist-van Holthe J.E., Ho P.L., Stablein D., Harmon W.E., Baum M.A. (2005). Outcome of renal transplantation for Wilms’ tumor and denys-drash syndrome: A report of the north American pediatric renal transplant cooperative study. Pediatr. Transplant..

[46] Grigoriev Y., Lange J., Peterson S.M., Takashima J.R., Ritchey M.L., Ko D., Feusner J.H., Shamberger R.C., Green D.M., Breslow N.E. (2012). Treatments and outcomes for end-stage renal disease following Wilms tumor. Pediatr. Nephrol..

